# ST-Segment Elevation Myocardial Infarction Secondary to Aortic Root Thrombosis in a Patient With a Left Ventricular Assist Device

**DOI:** 10.7759/cureus.116173

**Published:** 2026-09-13

**Authors:** Umama Gorsi, Julia Kumar, Nathan Kim, Pooneh Nabavizadeh, Naseer Khan

**Affiliations:** 1 Cardiology/Heart Failure, University System of Maryland, Baltimore, USA; 2 Internal Medicine, University of Cincinnati Medical Center, Cincinnati, USA; 3 Internal Medicine, Harbor-UCLA Medical Center, Torrance, USA; 4 Cardiology, University of Cincinnati Medical Center, Cincinnati, USA

**Keywords:** aortic root thrombus, destination therapy, left ventricular assist device (lvad), nicm, non-ischemic cardiomyopathy (nicm), st-elevation myocardial infarction (stemi)

## Abstract

Aortic root thrombosis (ART) is a rare but serious complication of continuous-flow left ventricular assist device (LVAD) support that can result in coronary artery occlusion. We present a 66-year-old man with a HeartMate 3 LVAD who presented with ST-segment elevation myocardial infarction (STEMI). Urgent coronary angiography revealed an aortic root thrombus with complete ostial right coronary artery (RCA) occlusion. He underwent successful percutaneous coronary intervention with drug-eluting stent placement. His course was complicated by ventricular fibrillation and right ventricular shock, followed by clinical recovery. This case highlights ART as an uncommon cause of acute myocardial infarction in LVAD-supported patients and emphasizes the importance of prompt recognition and management of ischemic complications in this population.

## Introduction

Left ventricular assist devices (LVADs) have become an integral component of the management of advanced heart failure, providing durable mechanical circulatory support as either a bridge to transplantation or destination therapy. Advances in continuous-flow LVAD technology, particularly with fully magnetically levitated centrifugal-flow devices, such as the HeartMate 3, have substantially improved survival, functional status, and quality of life in patients with advanced heart failure [1,2]. Despite these improvements, LVAD support remains associated with important complications, including bleeding, pump thrombosis, systemic thromboembolism, and ischemic events [1,2].

Aortic root thrombosis (ART) is an uncommon but potentially serious thrombotic complication of continuous-flow LVAD support. Continuous unloading of the left ventricle may reduce native left ventricular ejection and aortic valve opening, resulting in diminished antegrade flow through the aortic root [3,4]. Persistent or intermittent aortic valve closure can create areas of blood stasis within the aortic root and sinuses of Valsalva, providing a substrate for thrombus formation [3,4].

The clinical manifestations of ART vary from an incidental imaging finding to systemic embolization and potentially catastrophic coronary artery obstruction. Although uncommon, several reports have described ART extending into or obstructing a coronary ostium in patients receiving continuous-flow LVAD support, resulting in acute coronary syndromes and myocardial infarction [5-7]. Anticoagulation remains central to reducing thromboembolic complications during durable LVAD support, although balancing thrombotic and bleeding risks remains challenging [8-11]. We present a case of ST-segment elevation myocardial infarction (STEMI) caused by ART resulting in complete ostial right coronary artery (RCA) occlusion in a patient supported with a HeartMate 3 LVAD.

## Case presentation

A 66-year-old man with a history of nonischemic cardiomyopathy requiring HeartMate 3 LVAD implantation two years prior, chronic driveline infection with *Staphylococcus pseudintermedius*, atrial fibrillation, chronic kidney disease, and type 2 diabetes mellitus presented with acute-onset nausea, fatigue, diaphoresis, and left-sided neck pain. An electrocardiogram (ECG) demonstrated Q waves in the inferior leads and ST-segment elevations in the anteroseptal leads (Figure 1). High-sensitivity troponin was elevated at 3,897 ng/L.

**Figure 1:**
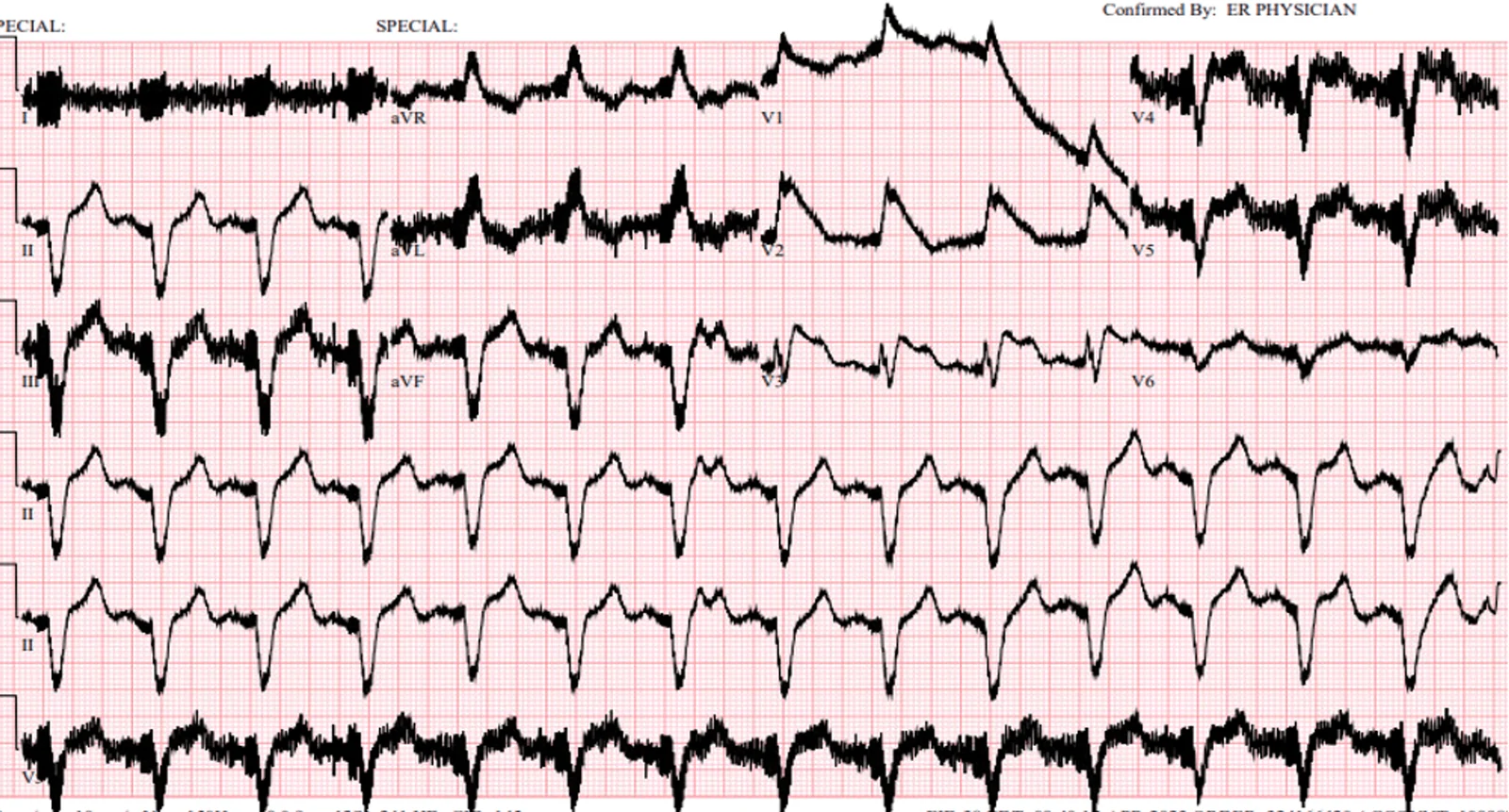
Patient's electrocardiogram (ECG) Normal sinus rhythm, rate of 85, nonspecific intraventricular block with prolonged QTc interval of 597 ms, left axis deviation, new ST elevations in leads V1-V5, significant baseline artifact from LVAD, and nonspecific Q waves in inferior leads.

The patient had previously been prescribed warfarin for LVAD anticoagulation but had a history of labile international normalized ratio (INR) values and missed doses. Because of these challenges, he had been transitioned to apixaban one month prior to presentation. At presentation, his anti-Xa level was mildly elevated at 1.31 U/mL (reference range: 0.5-1.2 U/mL). He received aspirin 324 mg, clopidogrel 600 mg, atorvastatin 80 mg, and heparin 4,000 units, and was taken for urgent coronary angiography.

Coronary angiography demonstrated a significant aortic root thrombus with complete occlusion of the ostial RCA (Figure 2A). The patient underwent successful percutaneous coronary intervention with placement of a drug-eluting stent in the ostial proximal RCA, with restoration of coronary flow on repeat angiography (Figure 2B).

**Figure 2:**
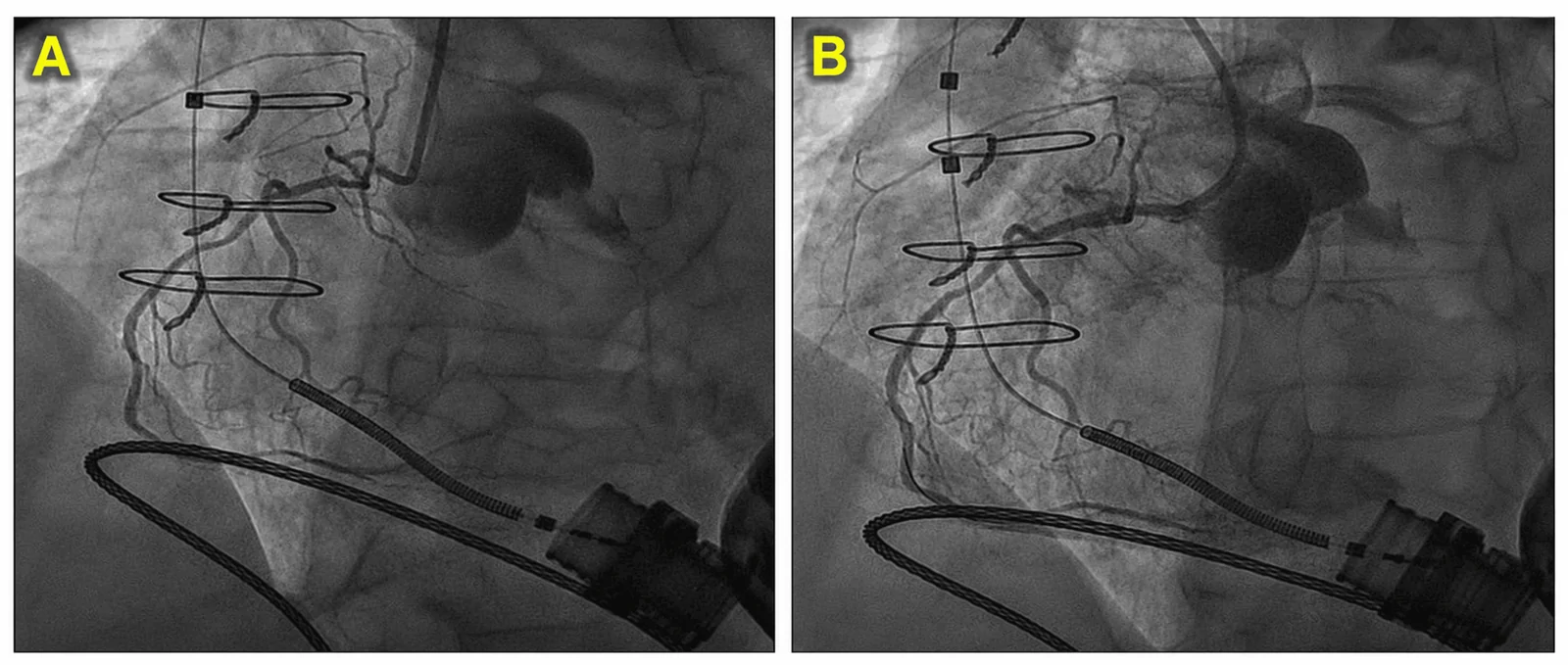
Coronary angiography before and after percutaneous coronary intervention (A) Coronary angiography demonstrating aortic root thrombus with complete occlusion of the ostial right coronary artery. (B) Repeat angiography following percutaneous coronary intervention and drug-eluting stent placement demonstrating restoration of right coronary artery flow.

During the procedure, the patient developed two episodes of ventricular fibrillation requiring cardioversion and amiodarone. Following the procedure, he was treated with clopidogrel and an 18-hour eptifibatide infusion. On hospital Day 2, he developed right ventricular shock requiring inotropic support with dobutamine and was subsequently intubated for acute hypoxemic respiratory failure. His clinical status gradually improved, and he was successfully weaned from inotropic support and extubated on hospital Day 10.

For long-term anticoagulation, he was transitioned from heparin to warfarin with a target INR of 2.0-3.0. His LVAD pump speed was increased to 5,000 rpm to optimize hemodynamics. Guideline-directed medical therapy, including metoprolol succinate 25 mg, valsartan 40 mg twice daily, and eplerenone 25 mg, was resumed as tolerated. The patient continued to improve and was discharged home on hospital Day 25 with close follow-up in the heart failure clinic.

## Discussion

Continuous-flow LVADs have significantly improved outcomes in patients with advanced heart failure. However, continuous-flow physiology alters normal aortic root hemodynamics and can predispose patients to thrombotic complications. By unloading the left ventricle, LVAD support reduces antegrade flow across the aortic valve, which may result in diminished or absent aortic valve opening [3,4]. Persistent aortic valve closure further promotes blood stasis within the aortic root and may contribute to the development of ART [4,5].

ART can have serious consequences, including systemic embolization and coronary ostial obstruction [2,5]. Although coronary artery involvement is uncommon, extension of an aortic root thrombus into a coronary ostium can result in acute myocardial infarction [5-7]. The 2023 Interagency Registry for Mechanically Assisted Circulatory Support (INTERMACS) annual report described myocardial infarction as an uncommon adverse event in LVAD-supported patients, with a reported incidence of approximately 0.2% [2]. In our patient, ART resulted in complete occlusion of the ostial RCA, leading to STEMI and requiring emergent percutaneous coronary intervention. This case demonstrates that clinically significant ART remains possible despite advances in contemporary continuous-flow LVAD technology.

The diagnosis of ART can be challenging because routine surveillance is not generally performed and clinical manifestations vary depending on thrombus location and associated complications. A high index of suspicion should therefore be maintained when an LVAD-supported patient presents with symptoms or findings concerning for myocardial ischemia or systemic embolization [5,8]. When coronary involvement is suspected, prompt diagnostic evaluation is particularly important because coronary ostial obstruction can result in myocardial infarction, ventricular arrhythmias, and hemodynamic deterioration, as demonstrated by the ventricular fibrillation and subsequent right ventricular shock observed in our patient.

Anticoagulation is an important component of preventing thromboembolic complications in patients with durable LVAD support. Our patient had previously received warfarin but experienced labile INR values and difficulty with adherence and had therefore been transitioned to apixaban one month before presentation. At admission, his anti-Xa level was mildly elevated at 1.31 U/mL. Although his prior anticoagulation history may represent a potential contributing factor, a direct causal relationship between anticoagulation status and the development of ART cannot be established in this case. ART is likely multifactorial, with altered aortic root hemodynamics, reduced native antegrade flow, and persistent aortic valve closure potentially contributing to thrombus formation. Following the acute event, the patient was transitioned from heparin to warfarin with a target INR of 2.0-3.0.

Although ART causing acute myocardial infarction has previously been reported in patients supported with continuous-flow LVADs, including the HeartMate 3, the present case adds to the limited literature by demonstrating complete ostial RCA obstruction requiring emergent percutaneous coronary intervention, complicated by intraprocedural ventricular fibrillation and subsequent right ventricular shock. The patient's initial presentation with predominantly atypical ischemic symptoms further illustrates the diagnostic challenge of acute coronary syndromes in LVAD-supported patients. Successful revascularization resulted in restoration of coronary flow and eventual clinical recovery despite these severe acute complications. This case therefore reinforces the importance of considering coronary ostial obstruction from ART when LVAD-supported patients develop otherwise unexplained ischemic symptoms, electrocardiographic abnormalities, ventricular arrhythmias, or acute hemodynamic deterioration.

## Conclusions

ART is a rare but potentially life-threatening complication of continuous-flow LVAD support that may cause coronary ostial obstruction and acute myocardial infarction. This case demonstrates complete ostial RCA occlusion requiring emergent percutaneous coronary intervention and highlights the potential for ventricular arrhythmias and right ventricular failure following acute coronary obstruction. Because ischemic presentations may be atypical in LVAD-supported patients, clinicians should maintain a high index of suspicion and pursue prompt diagnostic evaluation when new ischemic symptoms, electrocardiographic abnormalities, ventricular arrhythmias, or unexplained hemodynamic deterioration occur. Further studies are needed to better define strategies for the surveillance, prevention, and management of ART in patients receiving durable LVAD support.

## References

[1] Miller L, Birks E, Guglin M, Lamba H, Frazier OH (2019). Use of ventricular assist devices and heart transplantation for advanced heart failure. Circ Res.

[2] Jorde UP, Saeed O, Koehl D (2024). The Society of Thoracic Surgeons Intermacs 2023 annual report: focus on magnetically levitated devices. Ann Thorac Surg.

[3] Callington A, Long Q, Mohite P, Simon A, Mittal TK (2015). Computational fluid dynamic study of hemodynamic effects on aortic root blood flow of systematically varied left ventricular assist device graft anastomosis design. J Thorac Cardiovasc Surg.

[4] Patel H, Madanieh R, Kosmas CE, Vatti SK, Vittorio TJ (2015). Complications of continuous-flow mechanical circulatory support devices. Clin Med Insights Cardiol.

[5] Ilonze OJ, Torabi A, Guglin M, Saleem K, Rao R (2023). Aortic root thrombosis leading to STEMI in a Heartmate 3 patient. J Artif Organs.

[6] Kallur AS, Bien-Aime F, Sallam T, Jawaid Y, Zaaqoq AM (2023). Aortic root thrombus causing non-ST elevation myocardial infarction after left ventricular assist device. Perfusion.

[7] Tanna MS, Reyentovich A, Balsam LB (2017). Aortic root thrombus complicated by left main coronary artery occlusion visualized by 3D echocardiography in a patient with continuous-flow left ventricular assist device. Echocardiography.

[8] Bernhardt AM, Reichenspurner H (2024). Anticoagulation in LVAD recipients and thrombembolic complications: where do we stand? Where are we going to?. J Heart Lung Transplant.

[9] Shah P, Looby M, Dimond M (2024). Evaluation of the hemocompatibility of the direct oral anticoagulant apixaban in left ventricular assist devices: the DOAC LVAD study. JACC Heart Fail.

[10] Mhanna M, Ayyad M, Mortada I (2024). Direct oral anticoagulants versus warfarin in adults with durable left ventricular assist devices: a systematic review and meta-analysis. Curr Probl Cardiol.

[11] Shah P, Tantry US, Bliden KP, Gurbel PA (2017). Bleeding and thrombosis associated with ventricular assist device therapy. J Heart Lung Transplant.

